# Exploring Student Preconceptions of Readiness for Remote-Online Case-Based Learning: A Case Study

**DOI:** 10.2196/mededu.5348

**Published:** 2016-04-28

**Authors:** Peter Nicklen, Jennifer L Keating, Stephen Maloney

**Affiliations:** ^1^ Monash University – Peninsula Campus Department of Physiotherapy Melbourne Australia

**Keywords:** problem-based learning, professional education, teaching, distance education

## Abstract

**Background:**

Case-based learning (CBL) is an educational approach where students work in small, collaborative groups to solve problems. Web-conferencing software provides a platform to present information and share concepts that are vital to CBL. Previous studies have found that participants were resistant to change associated with implementing e-learning; however, strategies to reduce this resistance have not been explored.

**Objective:**

This study was designed to explore student preconceptions and understanding of remote-online case-based learning (RO-CBL).

**Methods:**

The study took place during the Bachelor of Physiotherapy program at Monash University, Victoria, Australia, in 2013. The entire third-year cohort (n=73) was invited to participate. The primary outcome of interest was students’ preconceptions of RO-CBL, collected via pre- and posttraining surveys.

**Results:**

Of the 73 students, 66 completed both surveys (attrition rate 9.6%). Three key themes relevant to student preconceptions of RO-CBL emerged: flexibility in time and location of CBL, readiness or hesitation to change to a Web-based format, and the value of training in RO-CBL that included a demonstration and trial run. Thirty-four percent of the participants were hesitant to move to an online format.

**Conclusions:**

This study explored students’ preconceptions of Web-based learning and evaluated the change in students’ attitudes after training. The results suggest that educational designers should not assume that students are confident and competent in applying these technologies to professional educational activities. By identifying students’ needs before implementation, training sessions can be designed to target these needs, and improve the understanding of RO-CBL and how it works in practice. This may reduce resistance to change, enhance students’ satisfaction, and ultimately improve the learning experience.

## Introduction

Case-based learning (CBL) is an educational approach where students work in small, collaborative groups to solve a series of problems that are presented in contexts similar to those in which they are likely to encounter them in practice [1]. In CBL, the learner is responsible for identifying knowledge deficits relating to the case. This encourages students to develop and manage learning goals and other strategies needed for lifelong learning [2]. Case-based learning typically involves face-to-face interaction with a focus on self-directed study [3].

Computer-assisted learning (CAL) is the implementation of computer technology to create a rich environment for active learning [4]. Key benefits of CAL are flexibility and accessibility, which promote student autonomy [1]. These characteristics have the potential to facilitate active self-directed learning, enhancing student knowledge and understanding [5]. Crawford [1] reviewed six qualitative investigations and concluded that the proposed benefits of CAL may complement the current CBL learning experience [1].

Four randomized controlled studies [6-9] concluded that Web-based CBL is comparable to face-to-face CBL with regard to student learning outcomes; however, none of these studies incorporated Web conferencing where students engage in a live activity with other students in real time. The synchronous communication, whiteboard, and screen-sharing functions of typical Web-conferencing software provide a platform to present information and share concepts [10]. These elements are at the core of the social constructivist pedagogy behind CBL and it is hypothesized that they could support the current CBL model. To the authors’ knowledge, no randomized controlled studies have been published evaluating Web-conferencing learning within CBL.

Preliminary results from our research team (personal communication by Stephen Maloney, via email, April 2, 2014) support the notion that Web-conferencing CBL also provides students with a learning experience that is comparable to face-to-face CBL. An important finding of this study was that participants reported low satisfaction with the Web-based activity and were challenged by the transition to the Web-based environment. Low student satisfaction has been reported by others regarding Web-based programs [8] and may occur because the learner is not adequately aware of how to operate effectively in an online platform [11]. Feelings of social isolation are also an important consideration when implementing Web-based activities. McInnerney and Roberts [12] suggest that minimizing social isolation may make the difference between a successful and an unsuccessful online learning environment for many students. Rheingold [13] stated that fear is an important element in novice computer users. Keller et al [14] reported that only a small minority of public health faculty are engaged in social media. Grajales III et al [15] suggested that fear of the unknown appears to be a major barrier to the adoption of social media and suggests this may be due to a lack of understanding. A review [16] evaluated barriers to effective e-learning and found that participants were resistant to change associated with implementing e-learning and had negative views of the value of e-learning.

Huang and McConnell [17] reported that perceived learning and course satisfaction are correlated. Leh [18] suggested that students’ preconceptions affect the way the students react to a situation, defining preconceptions as “conceptions that result from informal experiences in everyday life.” It is also suggested that these preconceptions can be very difficult to change [18]. Therefore, it is possible that negative preconceptions toward Web-based learning may account for poor student satisfaction. This highlights the importance of student satisfaction within their education and their preconceptions. Stromso et al [19] suggested that students’ level of computer skills and confidence may also influence their attitudes toward e-learning. Induction programs may need to be designed in response to assessed needs of a group of learners [11,16]. Hands-on training may help to facilitate learning particularly with students who are initially intimidated by Web-based learning [20]. However, this has not been formally evaluated.

This study was designed to explore student preconceptions and understanding of remote-online case-based learning (RO-CBL) as well as to identify training needs. The data were used to design training sessions that targeted these needs. It was hypothesized that implementing training designed to meet the learners’ needs may reduce negative preconceptions of Web-based CBL, improve the implementation process, and, subsequently, increase student satisfaction with the learning experience.

### Aims

#### Primary Aim

The primary aim of this study was to explore students’ preconceptions and understanding of RO-CBL.

#### Secondary Aims

The secondary aims were to explore student-reported training needs before the implementation of RO-CBL, as well as the reported effects of training on students’ preparedness.

## Methods

### Design

This study used a mixed method framework (qualitative and quantitative) whereby students were assessed, participated in training, and were then reassessed. In this single-cohort study, all participants were assessed on the same outcomes and exposed to the same intervention. Ethics approval for the study was obtained through the Monash University Human Research Ethics Committee (Ethics CF13/456 – 2013000200).

### Participants

This study took place during the first semester of the third year of the Bachelor of Physiotherapy program at Monash University, Victoria, Australia, in 2013. The entire third-year cohort (n=73) was invited to participate. This cohort had previously completed 4 semesters of face-to-face CBL and therefore understood the process of CBL. Case-based learning attendance is a compulsory component of the undergraduate program, and, therefore, all students had to participate in the RO-CBL and the training sessions to meet course requirements. An independent research assistant recruited participants through face-to-face delivery and distribution of an information package, which included the explanatory statement. Students who chose not to consent to participate in the study were not required to complete pre and post assessment measures related to the study.

### Context

In the Monash University Bachelor of Physiotherapy program, CBL is currently completed on campus in small groups of 4-6 students. Case-based learning is made up of Part 1 and Part 2, which are completed at the start and the end of the academic week, respectively. During Part 1, students are introduced to the case and are required to work through the subjective and objective examination. Learning issues are developed at the conclusion of Part 1, which students work on individually and present to their group in Part 2. During Part 2, several days later, students are required to discuss management and closure of the case. As part of this undergraduate degree, students are also required to travel between two campuses.

During semester 1 of the third year, students participated in RO-CBL. The learning activity took place over 1 week and required students to complete both Part 1 and Part 2 online. Unlike traditional face-to-face CBL, the RO-CBL allowed students to complete the learning activity at home, via Web-conferencing software, in groups of 4-6. One academic facilitator was responsible for logging in to all groups’ online chat rooms. “Google Hangouts” was the computer program used for the RO-CBL, because of the ease of access and convenience of the university’s licensing agreement. This was accessed in 2013. This allowed students to interact via webcam and microphone, as well as access and work on shared documents in “Google Docs.” The features used in the study were basic and common to most Web-conferencing software.

Training was provided to introduce students to the Web-conferencing software, allowing them to operate in the online environment and learn the steps required to successfully participate in RO-CBL. The training sessions made up the intervention part of this case study.

### Training (Intervention)

Two training sessions occurred 3 weeks before the RO-CBL. Students were required to attend a 60-minute information session that was run by the third-year co-coordinator. The coordinator was also the RO-CBL facilitator. During this session students were introduced to the RO-CBL and shown how to set up RO-CBL and use the key functions with a step-by-step demonstration of the Web-conferencing software. Students had access to the program in the weeks before the RO-CBL and were encouraged to explore it during this time. One week before the RO-CBL, students attended a 30-minute self-directed training session. Participants completed a checklist that involved setting up a RO-CBL environment and utilizing its key features. Two assistants were available to provide support as required.

### Outcomes

The primary outcome of interest was student preconceptions of RO-CBL and their reflection on these preconceptions after the training sessions. Secondary outcomes included students’ preconceptions of training requirements and response to training assessed. Data were collected via 2 paper-based surveys.

The first survey was completed before the first training session when students had not been introduced to RO-CBL. This survey explored students’ preconceptions of RO-CBL using questions 1-6 (Textbox 1) as well as an open-ended question (question 11). The response options for the Likert scales ranged from “strongly disagree” (1) to “strongly agree” (5). Students were also required to consider self-reported confidence using the Web-conferencing software and their training requirements, before being exposed to RO-CBL, on the same 5-point Likert scale (questions 7 and 8). Students were asked if they had previously participated in a video call or Web conference involving several people (questions 9-10). Finally, training requirements were also explored via an open-ended question (question 12).

**Pre- and posttraining survey questions.**

- Five-point Likert scale items with response options (Q1-8) 1=strongly disagree, 2=disagree, 3=neutral, 4=agree, 5=strongly agree:

1. I understand what a RO-CBL is, conducted via Web conference with file sharing
2. I understand how RO-CBL will work in practice
3. I could meet the CBL's learning objectives via RO-CBL
4. I could envisage RO-CBL being used in the future
5. I would like RO-CBL to be used in the future
6. I am looking forward to trialing RO-CBL
7. I am confident using Google documents
8. I am confident using Google hangouts

- Yes or no questions:

1. I have participated in a video-call, that is, webcam call before: yes/no.
2. I have participated in a Web conference before (ie, using a webcam for a conversation with more than one person): yes/no.

- Open-ended questions:

1. What are your thoughts on moving to RO-CBL?
2. What training do you think you would require to effectively participate in RO-CBL?

The second survey was distributed after the second training session once students had been exposed to RO-CBL. Students were required to complete the same questions scored on the same 5-point Likert scale as in the first survey (questions 1-8), allowing the comparison with their pretraining beliefs about RO-CBL and self-assessed confidence with the Web-conferencing software. This provided an indication of the efficacy of the training sessions. All outcomes were distributed and collected by an independent research assistant.

### Data Analysis

Responses to Likert scale items were presented in summary format, and pre- and posttraining session responses were compared and tested for statistical difference. A Kruskal-Wallis rank test was chosen as it can compare nonparametric data for 2 or more independent samples. Differences between pre- and posttest scores were considered statistically significant if the probability of differences occurring by chance alone was less than .05; Bonferroni adjustments were made to adjust significance levels for multiple questions. All quantitative statistical tests were performed using STATA 11 [21].

Responses to both open-ended questions (questions 11 and 12) were pooled, coded, and then themed using thematic analysis by 2 researchers working independently. This involved classifying and grouping segments of text to create and define themes that emerged from the data [22]. The emerged themes were then summarized and presented with supporting quotations. Responses to the open-ended question “What are your thoughts on moving to RO-CBL?” collected in the first survey were further categorized into either a positive or negative response. This was achieved via thematic analysis that, again, was completed by 2 researchers working independently. Likert responses that corresponded to the 2 positive and negative subgroups were then extracted and compared for differences in responses using a Kruskal-Wallis rank test.

## Results

### Participants

**Figure 1:**
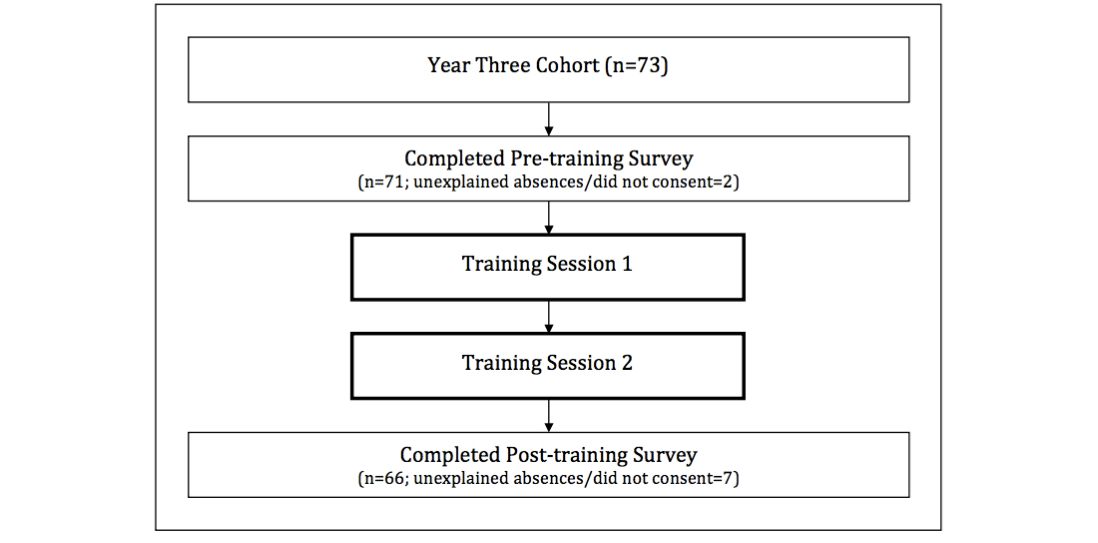
Participant flowchart and data collection points.

All 73 students enrolled in the third year of a Bachelor of Physiotherapy program at Monash University, Victoria, Australia, in 2013 were invited to participate. Of the 73 students, 54 were female and 19 were male. A total of 71 students completed the pretraining survey. Before the RO-CBL training, 61/71 students had participated in a video call and 27/71 had participated in a Web conference. All 73 students were required to attend the 2 training sessions. Of these, 66 students completed the posttraining survey (attrition rate 9.6%). Figure 1 summarizes participants’ flow and data collection process.

### Response to RO-CBL After Training Compared With Preconceptions

Participant responses to pre- and posttraining questionnaires are summarized in Table 1 and compared by Kruskal-Wallis rank test in Table 2.

After training, students were confident using the Web-conferencing software. They understood how RO-CBL worked in practice and felt they could meet CBL learning objectives.

**Table 1:** Summary data of pre- and posttraining questionnaire responses.

Item	Response^a^									
	1		2		3		4		5	
	Pre	Post	Pre	Post	Pre	Post	Pre	Post	Pre	Post
1. I understand what a RO-CBL^b^ is, conducted via a Web conference with file sharing	3	0	9	0	10	4	30	19	19	43
2. I understand how RO-CBL will work in practice	8	0	14	0	20	3	22	27	7	36
3. I could meet the CBL's^c^ learning objectives via RO-CBL	2	0	3	1	34	17	25	20	7	28
4. I could envisage RO-CBL being used in the future	4	1	6	4	20	15	30	19	11	27
5. I would like RO-CBL to be used in the future	6	3	8	4	32	28	17	19	8	12
6. I am looking forward to trialing RO-CBL	3	2	4	3	16	11	28	26	20	24
7. I am confident using Google documents	5	1	15	4	13	5	25	22	13	34
8. I am confident using Google hangouts	5	0	31	7	18	13	13	25	4	21

^a^Response options: 1=strongly disagree, 2=disagree, 3=neutral, 4=agree, 5=strongly agree.

^b^RO-CBL: remote-online case-based learning.

^c^CBL: case-based learning.

**Table 2:** Comparison of pre- and posttraining survey responses.

Item	Pretraining^a^ (n=71), median	Posttraining^a^ (n=66), median	Chi-square	*P*-value^b^
I understand what a RO-CBL^c^ is, conducted via a Web conference with file sharing	4	5	24.8	*<.001*
I understand how RO-CBL will work in practice	3	5	52.7	*<.001*
I could meet the CBL's^d^ learning objectives via RO-CBL	3	4	18.9	*<.001*
I could envisage RO-CBL being used in the future	4	4	7.6	*.006*
I would like RO-CBL to be used in the future	3	3	3.1	.08
I am confident using Google documents	4	5	21.8	*<.001*
I am confident using Google hangouts	2	4	35.8	*<.001*

^a^Key: 1=strongly disagree, 2=disagree, 3=neutral, 4=agree, 5=strongly agree.

^b^Italicized *P*-value=Bonferroni-adjusted statistically significant value, *P*≤.007.

^c^RO-CBL: remote-online case-based learning.

^c^CBL: case-based learning.

### Student Preconceptions of RO-CBL

Three key themes emerged from the open-ended questions of the pretraining survey: flexibility in time and location of CBL, readiness or hesitation to change to a Web-based format, and the value of training in RO-CBL that included a demonstration and trial run.

#### Theme 1: Flexibility

Students identified the flexibility that RO-CBL would provide to both students and the delivery of the physiotherapy course content.

I think it’s a good idea and will be great for future students to have a lot of the course online to offer more flexibility when we study.

An online format would allow students to complete CBL from home and therefore decrease travel to the university campus. Reducing travel lowers travel-related costs and allows students to allocate this time to alternative activities. Students reported valuing this extra time and money saved in petrol and road tolls.

I think it’s a good idea, will save a lot of time (transportation) it is very expensive to drive to Frankston and so avoiding that is a plus.

Students also noted the benefit of not having to travel between campuses to attend other classes on the same day. One student suggested that by completing CBL at home, communication with peers could be affected.

#### Theme 2: Change

Students were hesitant to test RO-CBL for a range of reasons. Seven students stated that they preferred face-to-face CBL before experiencing RO-CBL. Face-to-face enables participants to discuss the case in person, potentially allowing for greater interaction between students and a chance to develop interpersonal skills.

I prefer f2f [face-to-face] CBL so we can interact and feed off each other’s ideas more easily and use the whiteboard to help brainstorm.

Students were concerned that communicating with microphones and cameras would make conversations difficult.

Could be problematic with regards to computer failure and getting the webcam and mic set up and working.

Six students were concerned with other technology-related problems such as interruptions with the Internet connection. Nine students were looking forward to testing the RO-CBL, with one stating that they were “optimistic about its success.” Twelve students were unsure about how it would go but were “happy to try,” with 3 of these 12 stating they preferred face-to-face CBL.

Good idea in theory - haven’t had the practical experience yet to judge.

Potential benefits of RO-CBL perceived by the students included greater efficiency and easier collaboration once students adapt to the online learning environment.

#### Theme 3: Training Requirements

Two methods of training were recognized by the students—a demonstration and a trial run. Twelve students identified that the only training they would require was a demonstration or tutorial.

A step-by-step tute to understand the software, how to use it best.

The content of the demonstration proposed by students included how to conduct an RO-CBL, set up and check the microphone and camera are working, navigate the Web-conferencing software, invite people to the Web conference, and upload documents and share with group members. Other students suggested that a trial run would be sufficient. This would allow students to familiarize themselves with the program.

I think it would be fine, once we start doing it, it will click into place.

Students also expressed the need to troubleshoot issues before commencing the RO-CBL, regardless of the format. One student also suggested that more practice was required to concentrate and learn in an online environment.

I don’t think I will need much training in relation to the technical side of it, however maybe more practice in concentrating and learning through such means.

### Response to Open-Ended Questions Compared With Pretraining Likert Scales

The positive and negative responses to the open-ended question “What are your thoughts on moving to RO-CBL?” were assembled and compared using Likert responses to questions in the pretraining assessment responses (Table 3).

We found no significant relationship between confidence using the Web-conferencing program and preconceptions of RO-CBL. There were significant differences between those positively and negatively disposed toward RO-CBL to the questions “I would like RO-CBL to be used in the future” and “I am looking forward to trialing RO-CBL” in favor of positive responders, but not in responses to questions regarding use of the online medium.

**Table 3:** Pretraining assessment responses compared for those who wrote free-text responses that were classified as either positively or negatively disposed toward RO-CBL and compared using Kruskal-Wallis rank test (significance set at *P*<.008).

Item	Positive subgroup^a^ (n=39), median	Negative subgroup^a^ (n=20), median	Chi-square	*P*-value^b^
I understand what a RO-CBL^c^ is, conducted via a Web conference with file sharing	4	4	<0.01	.78
I understand how RO-CBL will work in practice	3	3	0.3	.59
I could meet the CBL's^d^ learning objectives via RO-CBL	4	3	1.2	.26
I could envisage RO-CBL being used in the future	4	3	4.9	.03
I would like RO-CBL to be used in the future	4	3	15.3	*<.001*
I am looking forward to trialing RO-CBL	4	3	8.8	*.003*
I am confident using Google documents	4	3	1.0	.31
I am confident using Google hangouts	2	2	2.4	.12

^a^Key: Response options 1=strongly disagree, 2=disagree, 3=neutral, 4=agree, 5=strongly agree.

^b^Italicized *P*value=statistically significant value.

^c^RO-CBL: remote-online case-based learning.

^d^CBL: case-based learning.

## Discussion

This study explored students’ preconceptions of Web-based learning and evaluated the change in their attitudes after training. Assumptions are frequently made about the information and communication technology literacy of students because of experience with, and accessibility to, Web-enabled devices. However, educational designers should not assume that students are confident and competent in applying these technologies to professional educational activities that require them to interact with their peers.

Approximately a third of the participants (23/71, 32%) did not agree with the statement “I am looking forward to trialing RO-CBL.” This hesitation to move to an online format may be due to a number of reasons. Before the training session, students reported that they understood how RO-CBL worked but were unsure how it would work in practice. They were unsure if learning objectives could be achieved in the online format, and, interestingly, 51% (36/71) of participants were not confident with the Web-conferencing software “Google Hangouts” before training. These factors might account for the hesitation to move to the online format.

There were several reasons identified by students that account for the apparent resistance to the adoption of RO-CBL including the risk of social isolation, potential technical difficulties, and anticipated difficulties with communication among CBL peers. Six participants (6/71, 8.5%) were concerned before training about potential technology-related problems such as interruptions with Internet connection. As students were able to complete CBL at home, they may not have been in the physical company of their peers, which might cause a perception of social isolation [23]. This was also recognized by Greenhalgh [4] who suggested that students may experience social isolation depending on their preferred style of learning or their stage in the development of online learning skills. Inadequate technology is an important concern for learners [16], and technical difficulties are commonly reported during Web-based programs [6,7,24]. This can also result in difficulties with communication [3,7,24]. This hesitation might not be unique to Web-based learning, but to change itself. Participants did, however, recognize that there could be benefits associated with moving to an online platform.

Flexibility is a commonly recognized benefit of Web-based learning—not only in relation to time, but also to travel demands [4,23]. Others have noted that the flexibility provided by Web-based learning applies to RO-CBL [3,24]. Participants in this study acknowledged that RO-CBL would reduce travel-related time and cost, which could be a possible motivator for adopting a Web-based format. Participants also recognized that RO-CBL may allow greater efficiency and easier collaboration once students adapted to the online learning environment. This finding was apparent in a similar study [24] where participants reported that even though the conferencing system was easy to use, a period of adaptation was experienced when moving to the online environment. Despite these identified benefits, students remained hesitant to move to a Web-based format before training.

Given this hesitation, we assume that some students may not be comfortable working in an online environment and therefore may require specific skill development. After the training sessions, there was a significant shift in participant responses to the Likert scales. Participants understood what RO-CBL was, how it would work in practice, how they could meet the learning objectives using this new mode of learning, and that they could see it used effectively in the future. Despite a positive shift toward the “strongly agree” end of the Likert response options, to “I would like RO-CBL to be used in the future,” this was not significant. Importantly, participants were also confident in the use of both “Google Docs” and “Google Hangouts.” This highlights the importance of targeted training sessions.

McLinden et al [11] found that participants with limited computer experience felt out of their depth when engaging with e-learning. However, we did not find a significant relationship between negativity toward Web-based learning and reported perceptions of ability. Regardless, it is still important that training is designed to meet the learner’s needs, as highlighted by Childs et al [16]. This ensures that training provides the required information technology skills to effectively learn in the Web-conference environment. Although most participants suggested a demonstration or tutorial would be sufficient, a small group of students expressed their preference to troubleshoot issues before testing the RO-CBL. This variation may again be due to different stages in the development of online learning skills or a sense that they needed to test their skills in real application to better anticipate obstacles that might be encountered. Greenhalgh [4] also suggested that students regularly use “just in time learning,” that is, only learning skills when they are immediately required. This may suggest theoretical training needs to be supported by training in real-time skill application.

### Limitations

Our participants had 2 years of previous face-to-face CBL experience, which means they understood how CBL worked in practice, even though they were new to the Web-based delivery. This may potentially reduce the transferability of our results with those less experienced with CBL. The validity and reliability of the surveys are unknown. However, the questions had face validity for gathering data on the construct of interest. This needs to be considered when interpreting our findings. Sample size was determined by students’ availability. We invited all available students in a single year level to participate in the study. For repeated measures analysis sample sizes of 30 or more are likely to provide normally distributed change scores. The sample was specific, as all participants had prior exposure to CBLs and were completing a course in which lecture content aligned with CBL content. The data obtained are therefore sample specific and the findings warrant validation in independent samples. Finally, one particular software program was used, and although only features common to other Web-conferencing programs were utilized, the transferability of results may still be affected.

### Conclusions

By identifying student needs before implementation, training sessions can be designed to target these needs, improving the understanding of RO-CBL and how it works in practice. This may reduce resistance to change when introducing Web-based learning, enhance student satisfaction with Web-based activities, and ultimately improve the learning experience. The findings of this research apply in the context of implementing a Web-conferenced RO-CBL to those already familiar with the CBL format.

On the basis of student-reported training needs and preconceived concerns, training might include how to log on and navigate the Web-conferencing software, how the CBL elements will be carried out in a Web-conference format, that is, what will change and what will remain the same, how to utilize functions of the Web-conferencing software required for RO-CBL, how to troubleshoot technical issues, and how to utilize synchronous and asynchronous communication methods.

## Abbreviations

CAL: computer-assisted learning
CBL: case-based learning
RO-CBL: remote-online case-based learning
